# Energy-delay analysis in advection-diffusion-based wireless body area networks

**DOI:** 10.1371/journal.pone.0330744

**Published:** 2025-08-29

**Authors:** Ghazaleh Kianfar, Pouya Hosseini, Mehdi Azadi, Jamshid Abouei, Arash Mohammadi

**Affiliations:** 1 Concordia Institute of Information Systems Engineering (CIISE), Concordia University, Montreal, Canada; 2 Department of Electrical Engineering, Yazd University, Yazd, Iran; Manipal Academy of Higher Education, INDIA

## Abstract

Molecular communication (MC) emerges as an encouraging concept in wireless body area nanonetworks (${\text{WBAN}}^{2}$), which utilizes molecules as information carriers for communication between nanomachines. In this paper, we aim to define an electrical model of a molecular-based nano-transmitter to analyze the effect of the remained transmitted molecules in a fluidic medium. To this end, we will address an advection-diffusion equation with a non-zero initial condition to analyze the residual molecules’ influence the medium. Moreover, considering the energy consumption limitations of nanomachines, we will employ the derived electrical model to further investigate how nanomachines consume the energy in presence of residual molecules. Following this, to enhance the energy consumption of the nano-transmitters, the settle-time method will be proposed to tackle the negative impact of the residual molecules on energy consumption. Nevertheless, since the proposed method increases the delay at nano-transmitters, the energy-delay trade-off relation at nano-transmitters will be investigated. Then, by introducing an interruption period and a control coefficient, we control the trade-off between the energy consumption and the created delay. Finally, by considering insulin molecules as messenger molecules in our simulations, we will demonstrate that implementing short interruption periods significantly enhances energy consumption, while introducing a small amount of delay to the system. Particularly, the energy consumption is reduced by 15% and the latency is increased by 2.2 ms when 1 ms interrupt period is used for 20 mol of insulin molecule.

## Introduction

Over the last decade, significant technological developments have revolutionized human healthcare systems. One prominent innovation in this area is the advent of wireless body area networks (WBANs), with various applications in remote patient emergency response, telemedicine, assisted living, and patient localization[1–6]. In this field, a nature-inspired communication paradigm, known as molecular communication (MC), has emerged in the realm of wireless body area nanonetworks (${\text{WBAN}}^{\text{2}}$s) [7–10]. Unlike traditional electromagnetic (EM)-based WBANs that rely on simple nano-structured sensors, MC employs nanomachines that release molecules as information carriers, enabling communication between transmitters and receivers. While EM-based systems primarily contend with thermal noise and channel fading, MC systems face distinct challenges such as molecular diffusion and inter-symbol interference (ISI) [11].

The nanoscale structure of nanomachines and the need to minimize propagation delays in various intra-body applications make energy consumption and delay two critical factors in MC systems. Therefore, analyzing the energy-delay trade-off in advection-diffusion-based MC nanonetworks presents a promising approach to enhance the functionality and practicality of these systems in healthcare. This paves the way for energy-delay optimization, leading to the reliability and responsiveness of these systems. This particularly improves the performance of time-sensitive applications such as insulin delivery, continuous glucose monitoring, and ECGs.

The problem of delay and energy consumption control in MC systems has been investigated in several papers in the literature [12–14]. In [12], the authors investigated the trade-off between rate and delay, employing a simple network coding mechanism to enhance the rate while considering the system’s delay. The study in [14] also proposed a energy control strategy based on feedback control theory. The need for continuous feedback from the receiver to the transmitter leads to higher energy consumption in the system. However, [12] and [14] neglected the additional energy the transmitter nanomachine consumes to adjust the transmission rate and energy, respectively. This issue becomes especially problematic under strict energy consumption restrictions to adapt the transmission rate. The authors in [13] demonstrated that if the nano-transmitter releases molecules faster than the receiver’s reaction, the excess molecules remaining in the medium degrade the system’s consumption. To address this issue, they proposed an approach wherein the receiver’s feedback controls the transmission rate. Although employing adaptive transmission rates improves energy consumption, continuous feedback from the receiver to the transmitter increases complexity in MC systems and incurs extra energy consumption to adjust the transmission rate. Therefore, in [15], the authors introduced a non-adaptive pre-equalization method to reduce inter-symbol interference (ISI) for binary concentration shift keying (BCSK)-encoded sequences in a single-transmitter single-receiver diffusive system. To this aim, they considered the difference between the number of received molecules of two different types of molecules as the actual signal at the receiver side. Nevertheless, leveraging various types of molecules for transmission introduces additional complexity in terms of molecular control, synthesis, and reception.

In the literature, several modulation schemes are explored for MC, including molecular type-based, time-based, and spatial domain-based modulations [16–18]. Nevertheless, given the importance of simplicity in nanomachines, we choose BCSK modulation with a fixed transmission rate, which is often desirable for sending bits in various real-world applications. Here, the term “fixed transmission rate” does not mean that the communication system employs a fixed rate for sending all the bits; rather, it refers to the release of a specific rate of molecule concentration by the nano-transmitter to modulate bit “1” and no molecules to represent bit “0”. However, when molecules are released at a fixed rate, residual molecules can accumulate, potentially leading to increased molecule loss and longer propagation delays.

The challenges outlined above inspired us to introduce a heuristic electrical model accounting for the presence of residual molecules in the medium and indicating their impact as a generated negative current towards the nano-transmitter. The main contributions of this paper are summarized as follows:

- We utilize the solved advection-diffusion channel equation from [19, 20] to formulate an electrical model for the channel, accounting for non-zero initial conditions. This model mitigates the degradation caused by residual molecules by incorporating a dependent current source. Additionally, we introduce a new parameter, termed the “effective rate”, which signifies the actual rate of released molecules that effectively diffuse through the channel from the transmitter to the receiver nanomachine. This parameter captures the influence of residual molecules by considering their resultant negative current directed toward the transmitter nanomachines. We then prove that the negative current generated during the transmission of the ${\text{n}}^{\text{th}}$ bit has a recursive relation with the negative currents caused by the preceding *n*–1 transmitted bits in previous time slots. It is demonstrated that the surplus of residual molecules leads to an exponential reduction in the effective rate.
- In the second part, we investigate how the remaining molecules influence the energy consumption and propagation delay. To this aim, we derive the energy model for the transmitter nanomachine by utilizing the mathematical expression of the channel with non-zero initial conditions. Subsequently, we demonstrate that the transmitter must exponentially increase the amount of released molecules through the medium to maintain a fixed transmission rate. It is also observed that the surplus of the released molecules correlates linearly with the energy consumption of the transmitter nanomachine.
- In the third section, we introduce an algorithm named “settle-time” to address the issue of exponential energy consumption escalation in the transmitter for sending *n* bits of information. This method primarily involves introducing interruptions between the transmission of each bit, leading to a reduction in the rate of the released molecules from the transmitter.
- Finally, we analyze the trade-off relationship between energy consumption and delay for transmission of a large number of bits. We illustrate that utilizing the relaxation time reduces the energy consumption of the transmitter at the expense of increased delay. Subsequently, we calculate and evaluate the optimal relaxation time that minimizes both energy consumption and propagation delay through simulations.

The rest of the paper is organized as follows: In section, we explain the assumptions and system model and provide the mathematical solution of the advection-diffusion equation under the non-zero initial condition. Section introduces an electrical model of the nano-transmitter in the presence of remaining molecules. The energy model of the nano-transmitter and the impact of residual molecules on its energy consumption are discussed in section. Then, we propose the “settle-time” method to enhance the energy consumption of the system in section. The trade-off relationship between energy consumption and the resulting delay at the nano-transmitter is analyzed in section. Finally, simulation results and conclusions are provided in section and section, respectively.

## System model and assumptions

In this section, we formulate the mathematical expression for the channel between the transmitter nanomachine (TN) and the receiver nanomachine, denoted by *NM*_*i*_ and *NM*_*j*_, respectively. We assume that the nanomachine *NM*_*i*_ modulates bits using the Binary Concentration Shift Keying (BCSK) modulation, similar to [7, 21, 22]. In addition, it is assumed that the nanomachine *NM*_*i*_ releases molecules at a fixed rate due to the energy consumption restrictions. Moreover, throughout this paper, the term “delay” refers to the delay incurred by propagating molecules from the transmitter to the receiver nanomachine. Thus, the delay of the transmitter and receiver circuits is neglected. To model the propagation channel from *NM*_*i*_ to *NM*_*j*_ during the transmission of ${\text{n}}^{\text{th}}$ bit, we apply the advection-diffusion expression in [7] as

$$\frac{\partial q_{ij}^{\left(n\right)}(x,t)}{\partial t}+R_{ij}q_{ij}^{\left(n\right)}(x,t)+u_{ij}(t)\frac{\partial q_{ij}^{\left(n\right)}(x,t)}{\partial x}\;\;-D_{ij}(t)\frac{\partial^{2}q_{ij}^{\left(n\right)}(x,t)}{\partial x^{2}}=0,$$ (1)

in which $q_{ij}^{\left(n\right)}(x,t)$ indicates the number of particles in each unit length of the path (*i*, *j*) when ${\text{n}}^{\text{th}}$ bit is released. Therefore, the number of molecules within a distance Δx from the TN is derived as $q_{ij}^{\left(n\right)}(x,t)\Delta x$. Additionally, the medium parameters *R*_*ij*_, *u*_*ij*_(*t*), and *D*_*ij*_(*t*) index the molecules’ degradation rate, the flow’s average velocity, and the medium’s diffusion coefficient, respectively. Therefore, the release rate of molecules through the path (*i*, *j*) during the transmission of ${\text{n}}^{\text{th}}$ bit is obtained by Fick’s Law as follows [23]:

$$J_{ij}^{\left(n\right)}(t)={\left[u_{ij}(t)q_{ij}^{\left(n\right)}(x,t)-D_{ij}(t)\frac{\partial q_{ij}^{\left(n\right)}(x,t)}{\partial x}\right]}_{x=0}.$$ (2)

Considering the quantity of molecules remaining from the past transmission along the path (*i*, *j*), we face zero or non-zero initial conditions. To solve Eq (1), we focus on the non-zero initial condition as we aim to analyze the impact of remaining molecules on energy consumption and transmission delay. Assuming that *u*_*ij*_(*t*) and *D*_*ij*_(*t*) remain fixed within each time slot *t* (i.e., $u_{ij}(t)=u_{ij}$ and $D_{ij}(t)=D_{ij}$), we express the advection-diffusion equation in Eq (1) in the Laplace domain as

$$(s+R_{ij})Q_{ij}^{\left(n\right)}(x,s)+u_{ij}\frac{\partial Q_{ij}^{\left(n\right)}(x,s)}{\partial x}\;\;-D_{ij}\frac{\partial^{2}Q_{ij}^{\left(n\right)}(x,s)}{\partial x^{2}}=q_{ij}^{\left(n\right)}(x,0),$$ (3)

where $Q_{ij}^{\left(n\right)}(x,s)$ represents the Laplace transform of $q_{ij}^{\left(n\right)}(x,t)$. Denoting $\Upsilon_{ij}^{\left(n\right)}(s)$ as the Laplace transform of $J_{ij}^{\left(n\right)}(t)$ in Eq (2), the rate at which molecule concentration is released from nanomachine *NM*_*i*_ through the path (*i*, *j*) for sending ${\text{n}}^{\text{th}}$ bit is obtained as follows:

$$\Upsilon_{ij}^{\left(n\right)}(s)={\left[u_{ij}Q_{ij}^{\left(n\right)}(x,s)-D_{ij}\frac{\partial Q_{ij}^{\left(n\right)}(x,s)}{\partial x}\right]}_{x=0}.$$ (4)

Then, the solution of the differential equation in Eq (1) under zero initial condition is obtained as

$$Q_{ij}^{\left(n\right)}(x,s)=A^{\left(n\right)}(s)e^{(g_{ij}+h_{ij}(s))\frac{x}{\ell_{ij}}}+\;B^{\left(n\right)}(s)e^{(g_{ij}-h_{ij}(s))\frac{x}{\ell_{ij}}},$$ (5)

where $\ell_{ij}$ symbolizes the length of the path (*i*, *j*), and ${\text{A}}^{\left(\text{n}\right)}(\text{s})$ and ${\text{B}}^{\left(\text{n}\right)}(\text{s})$ represent constant coefficients. In addition, we have $g_{ij}=\frac{u_{ij}\ell_{ij}}{2D_{ij}}$, $h_{ij}(s)=\frac{\alpha_{ij}(s)\ell_{ij}}{2D_{ij}}$, and $\alpha_{ij}(s)≜\sqrt{u_{ij}^{2}+4D_{ij}(s+R_{ij})}$. Clearly, the solution of Eq (1) under non-zero initial condition is obtained as

$$Q_{ij}^{\left(n\right)}(x,s)=A^{\left(n\right)}(s)e^{(g_{ij}+h_{ij}(s))\frac{x}{\ell_{ij}}}+\;B^{\left(n\right)}(s)e^{(g_{ij}-h_{ij}(s))\frac{x}{\ell_{ij}}}+f\;(x,s,q_{ij}^{\left(n\right)}(y,0)),$$ (6)

where the particular solution $f\;(x,s,q_{ij}^{\left(n\right)}(y,0))$ represents the quantity of residual molecules along the path (*i*, *j*) considering the non-zero initial condition $q_{ij}^{\left(n\right)}(y,0)$ after transmitting ${\text{n}}^{\text{th}}$ bit. The value of this particular solution is calculated as

$$f\;(x,s,q_{ij}^{\left(n\right)}(y,0))=\;\frac{e^{\left(g_{ij}-h_{ij}(s)\right)\frac{x}{\ell_{ij}}}}{\alpha_{ij}(s)}\int_{0}^{x}e^{\left(h_{ij}(s)-g_{ij}\right)\frac{y}{\ell_{ij}}}q_{ij}^{\left(n\right)}(y,0)dy-\;\frac{e^{\left(g_{ij}+h_{ij}(s)\right)\frac{x}{\ell_{ij}}}}{\alpha_{ij}(s)}\int_{0}^{x}e^{-(h_{ij}(s)+g_{ij})\frac{y}{\ell_{ij}}}q_{ij}^{\left(n\right)}(y,0)dy.$$ (7)

It should be noted that $f(0,s,q_{ij}^{\left(n\right)}(y,0))=0$ for ∀n. To find the solution for constants ${\text{A}}^{\left(\text{n}\right)}(\text{s})$ and ${\text{B}}^{\left(\text{n}\right)}(\text{s})$ in Eq (6), we apply the boundary conditions $Q_{ij}^{\left(n\right)}(0,s)$ and $Q_{ij}^{\left(n\right)}(\ell_{ij},s)$ as follows:

$$Q_{ij}^{\left(n\right)}(0,s)=A^{\left(n\right)}(s)+B^{\left(n\right)}(s)\;+f\;(0,s,q_{ij}^{\left(n\right)}(y,0))=A^{\left(n\right)}(s)+B^{\left(n\right)}(s)\equiv X_{ij}^{\left(n\right)}(s),$$ (8)

and

$$Q_{ij}^{\left(n\right)}(\ell_{ij},s)=A^{\left(n\right)}(s)e^{\left(g_{ij}+h_{ij}(s)\right)}+B^{\left(n\right)}(s)e^{\left(g_{ij}-h_{ij}(s)\right)}\;+f\;(\ell_{ij},s,q_{ij}^{\left(n\right)}(y,0))\equiv X_{ji}^{\left(n\right)}(s).$$ (9)

Then, the constants ${\text{A}}^{\left(\text{n}\right)}(\text{s})$ and ${\text{B}}^{\left(\text{n}\right)}(\text{s})$ are obtained as

$$A^{\left(n\right)}(s)=\frac{X_{ji}^{\left(n\right)}(s)e^{-g_{ij}}-X_{ij}^{\left(n\right)}(s)e^{-h_{ij}(s)}}{2\sinh(h_{ij}(s))}+\frac{\beta_{ij}^{\left(n\right)}(s)}{\alpha_{ij}(s)},$$ (10)

$$B^{\left(n\right)}(s)=\frac{X_{ij}^{\left(n\right)}(s)e^{h_{ij}(s)}-X_{ji}^{\left(n\right)}(s)e^{-g_{ij}}}{2\sinh(h_{ij}(s))}-\frac{\beta_{ij}^{\left(n\right)}(s)}{\alpha_{ij}(s)},$$ (11)

where

$$\beta_{ij}^{\left(n\right)}(s)\equiv \frac{-\alpha_{ij}(s)e^{-g_{ij}}}{2\sinh(h_{ij}(s))}f\;(\ell_{ij},s,q_{ij}^{\left(n\right)}(y,0)).$$ (12)

Substituting Eq (10) and Eq (11) into Eq (6), the number of passing molecules during the transmission of ${\text{n}}^{\text{th}}$ bit through the path (*i*, *j*) under the non-zero initial condition (i.e., $q_{ij}^{\left(n\right)}(y,0)\neq 0$) can be written as follows:

$$Q_{ij}^{\left(n\right)}(x,s)=\;\left(\frac{X_{ji}^{\left(n\right)}(s)e^{-g_{ij}}-X_{ij}^{\left(n\right)}(s)e^{-h_{ij}(s)}}{2\sinh(h_{ij}(s))}+\frac{\beta_{ij}^{\left(n\right)}(s)}{\alpha_{ij}(s)}\right)e^{(g_{ij}+h_{ij}(s))\frac{x}{\ell_{ij}}}\;+\left(\frac{X_{ij}^{\left(n\right)}(s)e^{h_{ij}(s)}-X_{ji}^{\left(n\right)}(s)e^{-g_{ij}}}{2\sinh(h_{ij}(s))}-\frac{\beta_{ij}^{\left(n\right)}(s)}{\alpha_{ij}(s)}\right)e^{(g_{ij}-h_{ij}(s))\frac{x}{\ell_{ij}}}\;+f\;(x,s,q_{ij}^{\left(n\right)}(y,0)).$$ (13)

We will employ Eq (13) to propose a novel electrical model for analyzing energy consumption and delay in a nano-transmitter under the non-zero initial condition.

## Proposed electrical model of nano transmitter

In this section, we introduce an electrical model of the molecule transmission through the advective-diffusive medium under the non-zero initial condition. To this end, we initially define the concentration of molecules during the release of ${\text{n}}^{\text{th}}$ bit as follows:

$$C_{i}^{\left(n\right)}(s)=\frac{X_{ij}^{\left(n\right)}(s)}{S_{ij}}\;\text{and}\;C_{j}^{\left(n\right)}(s)=\frac{X_{ji}^{\left(n\right)}(s)}{S_{ij}},$$ (14)

where *S*_*ij*_ denotes the cross-sectional area of the path (*i*, *j*). In addition, $C_{i}^{\left(n\right)}(s)$ and $C_{j}^{\left(n\right)}(s)$ represent the molecular concentrations at *x* = 0 and $x=\ell_{ij}$ in the Laplace domain, respectively. By substituting Eq (13) and Eq (14) into Eq (4), concerning $f(0,s,q_{ij}^{\left(n\right)}(y,0))=0$ for all initial conditions, the rate at which molecules are released is obtained as

$$\Upsilon_{i}^{\left(n\right)}(s)=S_{ij}(\frac{u_{ij}}{2}+\frac{\alpha_{ij}(s)}{2\tanh(h_{ij}(s))})C_{i}^{\left(n\right)}(s)\;\;-S_{ij}\frac{\alpha_{ij}(s)e^{-g_{ij}}}{2\sinh(h_{ij}(s))}C_{j}^{\left(n\right)}(s)-\beta_{ij}^{\left(n\right)}(s).$$ (15)

**Fig 1:**
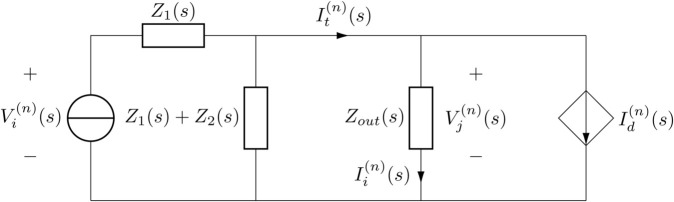
An equivalent electrical circuit model of nano-transmitter.

It should be noted that we omitted the subscript “*j*” at the boundary *x* = 0 for notation simplification and replaced $\Upsilon_{ij}^{\left(n\right)}(s)$ with $\Upsilon_{i}^{\left(n\right)}(s)$. To provide an analytical tool for representing the molecular communication process in the advection-diffusion, $\Upsilon_{i}(n)(s)$ and *C*_*j*_(*n*)(*s*) are mapped to the electrical current *I*_*i*_(*n*)(*s*) and voltage $V_{j}(n)(s)$, respectively, and we define voltage $V_{i}^{\left(n\right)}(s)=C_{i}^{\left(n\right)}(s)$  +  $2C_{j}^{\left(n\right)}(s)$. Additionally, the mathematical terms corresponding to channel properties in Eq (15), i.e., medium velocity, diffusion coefficient, and path length, can be represented as circuit impedances *Z*_1_(*s*) and *Z*_2_(*s*). As a result, the equivalent electrical circuit model of nano-transmitter *NM*_*i*_ is introduced in Fig 1, where the values of *Z*_1_(*s*) and *Z*_2_(*s*) are obtained as

$$Z_{1}(s)=S_{ij}(\frac{u_{ij}}{2}+\frac{\alpha_{ij}(s)}{2\tanh(h_{ij}(s))}),$$ (16)

$$Z_{2}(s)=S_{ij}\frac{\alpha_{ij}(s)e^{-g_{ij}}}{2\sinh(h_{ij}(s))}.$$ (17)

Additionally, *Z*_*out*_(*s*) and $I_{t}^{\left(n\right)}(s)$ refer to the external resistance of the channel and the total current before subtracting the negative current, respectively.

**Created adverse current:** The adverse current $I_{d}^{\left(n\right)}(s)$ models $\beta_{ij}^{\left(n\right)}(s)$, representing the effect of ISI created by the residual molecules from transmitting (*n*–1)^th^ bit during the ${\text{n}}^{\text{th}}$ time slot. Therefore, the quantity of remaining molecules that existed through the medium during sending ${\text{n}}^{\text{th}}$ bit, which denotes the relationship between the adverse current and the residual molecules, is expressed as

$$N_{d}^{\left(n\right)}=\int_{(k-1)T_{n}}^{nT_{n}}{ℒ}^{-1}\{I_{d}^{\left(n\right)}(s)\}dt,$$ (18)

where *T*_*n*_ denotes the transmission duration, and $\phi_{d}^{\left(n\right)}(t)={ℒ}^{-1}\{I_{d}^{\left(n\right)}(s)\}$. Then, the total adverse current during the transmission of the ${\text{n}}^{\text{th}}$ bit is calculated by accounting for the adverse current created by sending previous bits as

$$I_{d_{total}}^{\left(n\right)}(s)=\sum_{k=1}^{n-1}I_{d}^{\left(k\right)}(s).$$ (19)

**Fig 2:**
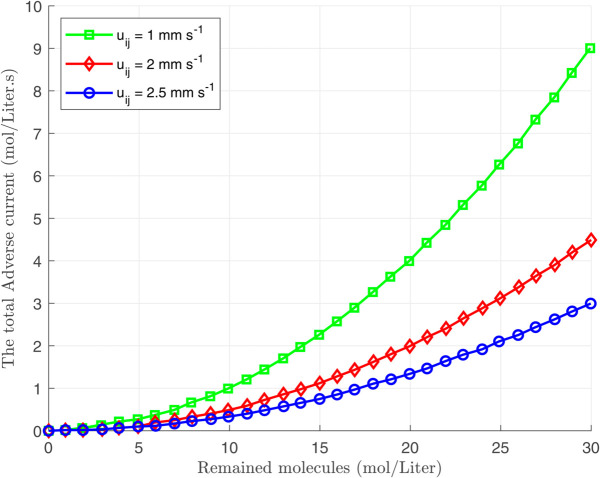
Created adverse current through the path after sending ${\text{n}}^{\text{th}}$ bit.

As depicted in Fig 1, the presence of remaining molecules through the medium reduces the output current $I_{i}^{\left(n\right)}(s)$ by generating adverse current $I_{d_{total}}^{\left(n\right)}(s)$. As a result, the actual rate of molecules propagating through the medium becomes lower than the rate of released molecules from the transmitter nanomachine. Therefore, to represent the actual rate of diffused molecules through the channel, we define the effective rate ${ℛ}_{eff}^{\left(n\right)}(t)$ as follows:

$${ℛ}_{eff}^{\left(n\right)}(t)≜{ℒ}^{-1}\{I_{t}^{\left(n\right)}(s)-I_{d_{total}}^{\left(n\right)}(s)\},$$ (20)

where $I_{i}^{\left(n\right)}(s)$ denotes the rate of released molecules from the transmitter nanomachine for sending ${\text{n}}^{\text{th}}$ bit. As observed from Eq (20), the effective rate of diffused molecules toward the nano-receiver decreases by increasing $I_{d_{total}}^{\left(n\right)}(s)$. Moreover, as illustrated in Fig 2, the adverse current $I_{d_{total}}^{\left(n\right)}(s)$ monotonically increases with the accumulation of molecules within the medium. Therefore, to maintain a fixed effective rate, it is necessary to steadily increase the rate of released molecules from the nano-transmitter to counteract the effect of $I_{d_{total}}^{\left(n\right)}(s)$. To this end, the transmitter is required to release additional molecules for transmitting ${\text{n}}^{\text{th}}$ bit as

$$N_{d_{total}}^{\left(n\right)}=\sum_{k=1}^{n}\int_{(k-1)T_{n}}^{nT_{n}}{ℒ}^{-1}\{I_{d}^{\left(n\right)}(s)\}dt,$$ (21)

To demonstrate how the presence of residual molecules affects the energy consumption of the transmitter nanomachine, we investigate the energy model of the nano transmitter in the next section.

## An energy model of nano-transmitter

**Fig 3:**
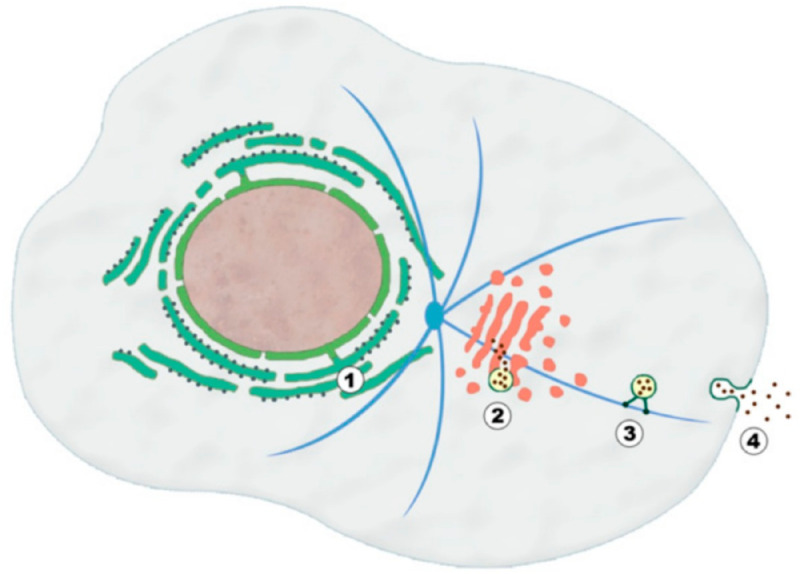
Four phases of exocytosis for preparing molecules to release through the medium[27].

In this part, we model the energy consumption of the transmitter nanomachine, inspired by the procedure utilized by eukaryotic cells [24–27]. In molecular cell biology, this procedure is known as exocytosis, encompassing four phases as follows (see Fig 3) [28]:

(1) Synthesis of the messenger molecules from their building blocks.
(2) Production of a secretory vesicle.
(3) Carrying the secretory vesicles to the cell membrane.
(4) Releasing the molecules via the vesicle and cell membrane fusion.

To calculate the total energy consumption *E*_*total*_, we denote the energy consumed during these four phases as *E*_*s*_, *E*_*p*_, *E*_*c*_, and *E*_*e*_, respectively. Therefore, the total energy consumed by the transmitter nanomachine for sending *N* molecules through the medium is obtained as

$$E_{total}=NE_{s}+\lceil \frac{N}{N_{c}}\rceil (E_{p}+E_{c}+E_{e}),$$ (22)

where ⌈.⌉ represents the ceiling operator, and *N*_*c*_ indexes the number of carried messenger molecules through each vesicle. By utilizing protein molecules as messengers, the synthesis energy refers to the energy required for forming peptide bonds between amino acids. In this case, the energy consumed for forming a bond between two amino acids is constant and equals 202.88 (zJ), where zeptojoule (zJ) is 10^−21^ Joules [29]. Therefore, the energy consumed for synthesizing one protein molecule composed of *N*_*aa*_ amino acids is $E_{s}=202.88(N_{aa}-1)\;\text{zJ}$. Similarly, the energy required for producing one vesicle mostly composed of phospholipids equals 83 zJ [30]. Hence, the energy of producing vesicles is *E*_*p*_ = 83 × $5(4\pi {r_{v}}^{2})\;\text{zJ}$, where $r_{v}$ denotes the radius of each vesicle. In addition, $E_{e}=83.10\;\text{zJ}$ and the value of *E*_*c*_ can be obtained as $E_{c}=83\lceil \frac{r_{unit}}{16}\rceil \;\text{zJ}$, where *r*_*unit*_ represents the radius of the transmitter nanomachine [28,31]. Taking the above considerations into account, the amount of energy consumption in the nano-transmitter for sending the ${\text{n}}^{\text{th}}$ bit is obtained as:

$$E_{T}^{\left(n\right)}=202.88(N_{aa}-1)(N+N_{d}^{\left(n\right)})+\lceil \frac{N+N_{d}^{\left(n\right)}}{N_{c}}\rceil \;\;\times (83\times 5(4\pi {r_{v}}^{2})+83\lceil \frac{r_{unit}}{16}\rceil +83.10)\;\text{zJ}.$$ (23)

Considering Eq (23), we notice that the total energy consumption at the transmitter changes linearly with $N_{d}^{\left(n\right)}$. In addition, we observed in section [electrical model] that the adverse current monotonically increases the number of remaining molecules in the medium. Consequently, the adverse current increases the energy consumption of the nano-transmitter exponentially. Therefore, a practical solution for minimizing the energy consumption at the nano-transmitter, named “settle-time method”, is proposed to reduce the adverse effect of the remaining molecules.

## Proposed settle time method to enhance energy consumption

In this section, we propose an approach to minimize the energy consumption in the nano-transmitter through analyzing the variation of adverse current over time. To obtain the adverse current in the time domain, we employ the inverse Laplace transform of $\beta_{ij}^{\left(n\right)}(s)$, defined in Eq (12) as follows:

$$\phi_{d_{total}}^{\left(n\right)}(t)={ℒ}^{-1}\{\sum_{k=1}^{n-1}I_{d}^{\left(k\right)}(s)\}={ℒ}^{-1}\{\sum_{k=1}^{n-1}\beta_{ij}^{\left(k\right)}(s)\}\;={ℒ}^{-1}\{\sum_{k=1}^{n-1}\frac{-\alpha_{ij}(s)e^{-g_{ij}}}{2\sinh(h_{ij}(s))}f\;(\ell_{ij},s,q_{ij}^{\left(k\right)}(y,0))\}.$$ (24)

**Fig 4:**
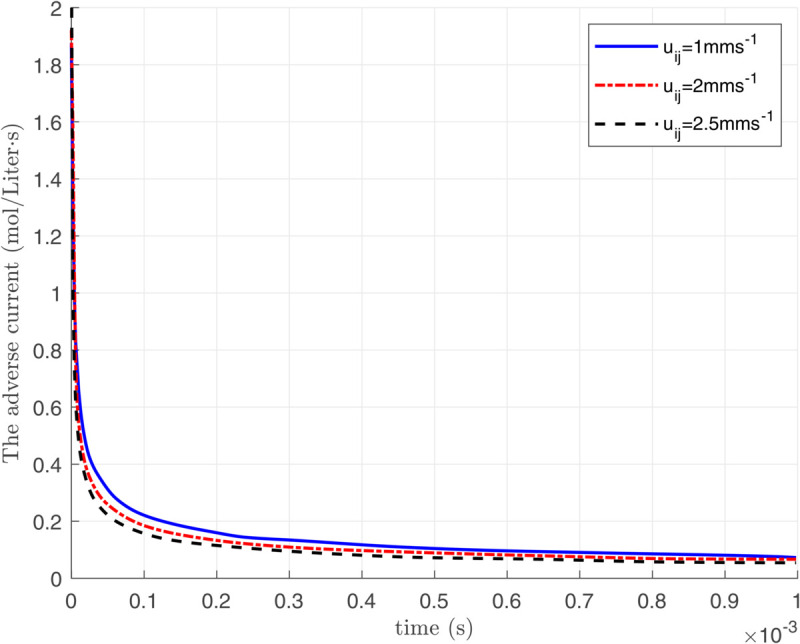
Created adverse current after sending bit 1.

**Fig 5:**
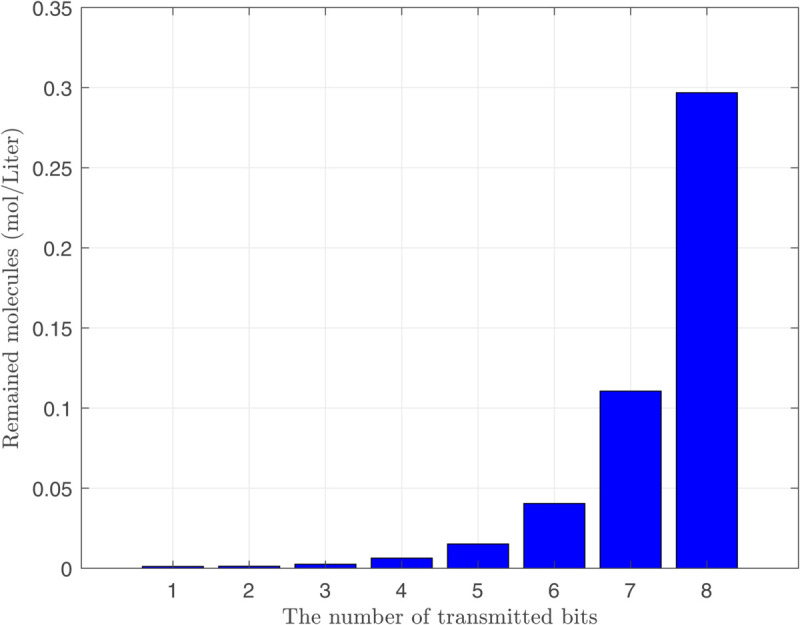
The effect of each transmitted bit on creating remained molecules through the medium at time of transmitting $9^{\text{th}}$ bit.

Due to the complexity of calculating a closed-form expression for the adverse current $\phi_{d_{total}}^{\left(n\right)}(t)$, Eq (24) is numerically analyzed and depicted in Fig 4. As observed, the impact of the adverse current created from sending bit 1 reduces as time passes. Similarly, we can conclude that the number of remaining molecules is also a decreasing function of time. Therefore, the number of residual molecules during the ${\text{n}}^{\text{th}}$ time interval is primarily influenced by the molecules transmitted in the more recent time slots. As depicted in Fig 5, all transmitted bits before the transmission of $9^{\text{th}}$ bit have different effects on the amount of residual molecules during the transmission of $9^{\text{th}}$ bit. Therefore, we ignore the effect of the previously transmitted bits with negligible impact on creating residual molecules to simplify the calculations. To this aim, we define $N_{d_{\tau }}^{\left(n\right)}$ as the minimum value of the remaining molecules that have a considerable effect on the effective transmission rate of the ${\text{n}}^{\text{th}}$ bit. Furthermore, we define the counting function of the considerable remaining molecules as follows:

$$f_{c}^{\left(k\right)}=\left\{ \begin{array}{cc} 1, & N_{d}^{\left(k\right)}\geq N_{d_{\tau }}^{\left(n\right)}\\ 0, & N_{d}^{\left(k\right)}<N_{d_{\tau }}^{\left(n\right)}, \end{array}\right.$$ (25)

where 1 ≤ *k* ≤ *n* − 1. Then, the number of bits $B_{eff}^{\left(n\right)}$ with considerable effect on the effective transmission rate of ${\text{n}}^{\text{th}}$ bit is obtained as

$$B_{eff}^{\left(n\right)}=\sum_{k=1}^{n-1}f_{c}^{\left(k\right)}.$$ (26)

Therefore, using (??), Eqs (19), (??), and (??) can be written as

$$I_{d_{eff}}^{\left(n\right)}(s)=\;\left\{ \begin{array}{cc} \sum_{k=n-B_{\text{eff}}^{\left(n\right)}}^{n-1}I_{d}^{\left(k\right)}(s), & B_{eff}^{\left(n\right)}\neq 0\\ 0, & B_{eff}^{\left(n\right)}=0 \end{array}\right.,$$ (27)

$${ℛ}_{eff}^{\left(n\right)}={ℒ}^{-1}\{I_{t}^{\left(n\right)}(s)-I_{d_{eff}}^{\left(n\right)}(s)\}{|}_{t=nT_{n}},$$ (28)

$$N_{d_{eff}}^{\left(n\right)}=\sum_{k=n-B_{\text{eff}}}^{n}\int_{(k-1)T_{n}}^{nT_{n}}\phi_{d}^{\left(n\right)}(t)dt.$$ (29)

Thus, the energy consumption of the nano-transmitter is approximated as follows:

$$E_{T}^{\left(n\right)}\approx 202.88(N_{aa}-1)(N+N_{d_{eff}}^{\left(n\right)})+\lceil \frac{N+N_{d_{eff}}^{\left(n\right)}}{N_{c}}\rceil \;\;\times (83\times 5(4\pi {r_{v}}^{2})+83\lceil \frac{r_{unit}}{16}\rceil +83.10)\;\text{zJ}.$$ (30)

Eq (30) demonstrates that the practical energy consumption at the nano-transmitter for sending the ${\text{n}}^{\text{th}}$ bit depends on the exocytosis phases for releasing the ${\text{n}}^{\text{th}}$ bit and $N_{d_{eff}}^{\left(n\right)}$ resulting from transmitting previous bits. Therefore, to decrease the energy consumption of the nano-transmitter, we define the settle-time *T*_*s*_ to apply an interruption between each two consecutive bits as

$$T_{s}\;:\;\phi_{d_{total}}^{\left(n\right)}(t){|}_{t=n(T_{n}+T_{s})}=\zeta \times \max\{\phi_{d_{total}}^{\left(n\right)}(t)\},$$ (31)

where  0<ζ<1 indexes the control coefficient of the amount of the adverse current remaining through the medium after transmitting the ${\text{n}}^{\text{th}}$ bit. In other words, changing ζ controls the value of $B_{eff}^{\left(n\right)}$, which also impacts the total energy consumption of the nano-transmitter, as shown in Eq (23). By applying interruption *T*_*s*_ between each two successive bit transmissions, we have

$${ℛ}_{s}^{\left(n\right)}={ℒ}^{-1}\{I_{t}^{\left(n\right)}(s)-I_{d_{eff}}^{\left(n\right)}(s)\}{|}_{t=n(T_{n}+T_{s})},$$ (32)

$$N_{d_{s}}^{\left(n\right)}=\sum_{k=n-B_{\text{eff}}}^{n}\int_{\left(k-1)(T_{n}+T_{s}\right)}^{n(T_{n}+T_{s})}\phi_{d}^{\left(n\right)}(t)dt,$$ (33)

where ${ℛ}_{s}^{\left(n\right)}$ and $N_{d_{s}}^{\left(n\right)}$ represent effective rate and the number of remaining molecules after applying the interruption *T*_*s*_, respectively. Consequently, the energy consumption of the transmitter nanomachine is approximated as

$$E_{T_{s}}^{\left(n\right)}\approx 202.88(N_{aa}-1)(N+N_{d_{s}}^{\left(n\right)})+\lceil \frac{N+N_{d_{s}}^{\left(n\right)}}{N_{c}}\rceil \;\;\times (83\times 5(4\pi {r_{v}}^{2})+83\lceil \frac{r_{unit}}{16}\rceil +83.10)\;\text{zJ}.$$ (34)

Eq (34) indicates that the optimum value of $E_{T_{s}}^{\left(n\right)}$ occurs when the number of remaining molecules goes toward zero (i.e., $N_{d_{s}}^{\left(n\right)}\to 0$). This situation happens when $B_{eff}^{\left(n\right)}=0$ and ζ→0. This will result in a too-large *T*_*s*_, which is impractical for many real-time applications. Therefore, it is necessary to choose a proper value for ζ to have efficient energy consumption and delay. To this end, we will investigate the trade-off between energy consumption and delay via controlling ζ in the next section.

## Energy-delay analysis at nano-transmitter

In this section, we analyze the trade-off between the energy consumption and created delay as a result of introduced interruptions. Depending on the nature of the application, we should tune the value of the parameter ζ. For example, the delay is more important than the energy consumption in real-time applications. However, in case we have restrictions on the consumed energy, we can set lower values to ζ and tolerate a larger delay. To this end, we initially define the delay of sending the ${\text{n}}^{\text{th}}$ bit as follows:

$$\tau_{D}^{\left(n\right)}=\tau_{c}^{\left(n\right)}+\tau_{p}^{\left(n\right)},$$ (35)

where $\tau_{c}^{\left(n\right)}$ and $\tau_{p}^{\left(n\right)}$ denote the delay resulted from the four phases of exocytosis and the propagation delay for sending the ${\text{n}}^{\text{th}}$ bit, respectively. Considering Eq (31), the total amount of delay for transmitting a packet with the length *N*_*p*_ is calculated as

$$\tau_{total}=\sum_{n=1}^{N_{p}}\tau_{D}^{\left(n\right)}+N_{p}T_{s}.$$ (36)

Given that the adverse current $\phi_{d_{total}}^{\left(n\right)}(t)$ is a monotonically decreasing function over time (see Fig 4), increasing the value of ζ in Eq (36) reduces the total delay of the nano-transmitter. Conversely, as indicated in Eq (31), an increase in ζ shrinks the period *T*_*s*_, leading to higher values of the adverse current $\phi_{d_{total}}^{\left(n\right)}(t)$. As discussed, although decreasing *T*_*s*_ reduces the delay, this increases energy consumption, indicating an inherent trade-off between energy consumption and delay.

**Remark 1:** Based on the above arguments, there exists a linear relation between ζ and the consumed energy, whereas, any variation in the value of *T*_*s*_ exponentially impacts the energy consumption, as will be shown in section [simulation].

## Numerical results

In this section, we numerically evaluate the energy consumption of the nano-transmitter and the created delay for sending a data packet from a nano-transmitter toward a nano-receiver. The nano-transmitter modulates the desired information to carrier molecules and prepares them in four phases of exocytosis to release them through the advective-diffusive medium. The values in Table 1 represent the physical characteristics of the nano-transmitter and the medium for insulin propagation as information carrier molecules. Since it is generally unrealistic to assume that the medium is completely free of residual molecules, all experiments in this work are conducted under non-zero initial conditions to more accurately represent practical molecular communication environments, where the presence of remaining molecules from prior transmissions is inevitable. In our work, the mathematical formulation and electrical modeling are parameterized with respect to the properties of the messenger molecule (see Eqs (1)–(7) and Table 1), and can therefore be readily adapted for other molecular species. It is worth noting that the simulations only focus on insulin as the messenger molecule, following our previous works [7, 34]. To analyze the discussed nanonetwork, we consider various scenarios for our simulations as follows:

- **Scenario 1:** In this scenario, we illustrate the changes in the energy consumption $E_{T_{s}}^{\left(n\right)}$ for different values of remaining molecules $N_{d_{s}}^{\left(n\right)}$ based on Eq (34). Furthermore, we consider different values for *T*_*s*_ and ζ to evaluate the influence of the settle-time method on reducing the energy consumed at the nano-transmitter.
- **Scenario 2:** In this scenario, we demonstrate how utilizing the settle-time method affects the delay at the nano-transmitter during the transmission of a data packet with *N*_*p*_ bits. To this end, we consider different values of *T*_*s*_ and ζ in (36) and plot the total created delay at the nano-transmitter.
- **Scenario 3:** In the next step, the relationship between the total energy consumption and the created delay at the nano-transmitter is investigated. In this regard, we calculate the value of $N_{d_{s}}^{\left(n\right)}$ for a specific amount of consumed energy using Eq (34). Then, we numerically analyze the impact of the parameter ζ and determine the corresponding created delay for a given value of energy consumption using Eq (36).

**Table 1:** The physical characteristics values of the path (*i*, *j*) and the nano-transmitter.

Physical Parameter	Value
Medium velocity of the path (*i*, *j*) (*u*_*ij*_)	2 mm/s
Length of the path (*i*, *j*) ($\ell_{ij}$)	1 mm
Cross sectional area of the path (*i*, *j*) (*S*_*ij*_)	1000 $\mu {\text{m}}^{2}$
Rate of insulin molecules lost or consumed along the path (*i*, *j*) (*R*_*ij*_)	1.58 ${\text{s}}^{-1}$ [7]
Transmission period of one bit (*T*_*n*_)	8 ms
Propagation delay ($\tau_{p}$)	3 ms
Exocytosis phases delay ($\tau_{c}$)	1 ms
Diffusion coefficient of the path (*i*, *j*) (*D*_*ij*_)	10^−6^ ${\text{m}}^{2}/\text{s}$ [32]
Number of molecules at each bit transmission (*N*)	100
Number of amino acids (*N*_*aa*_)	51
Number of packet bits (*N*_*p*_)	8
Radius of the vesicle ($r_{v}$)	0.5μm [33]
Radius of the nano-transmitter (*r*_*unit*_)	10μm [28]
Frequency (*f*)	0.1 KHz [32]

**Fig 6:**
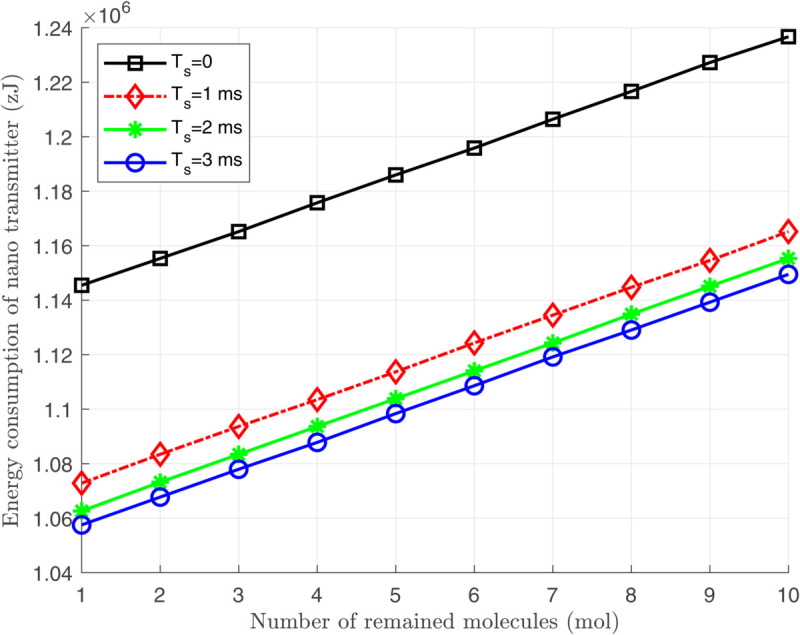
The total energy consumption *E*_*T*_ at the nano-transmitter in terms of the number of remaining molecules through the medium.

**Fig 7:**
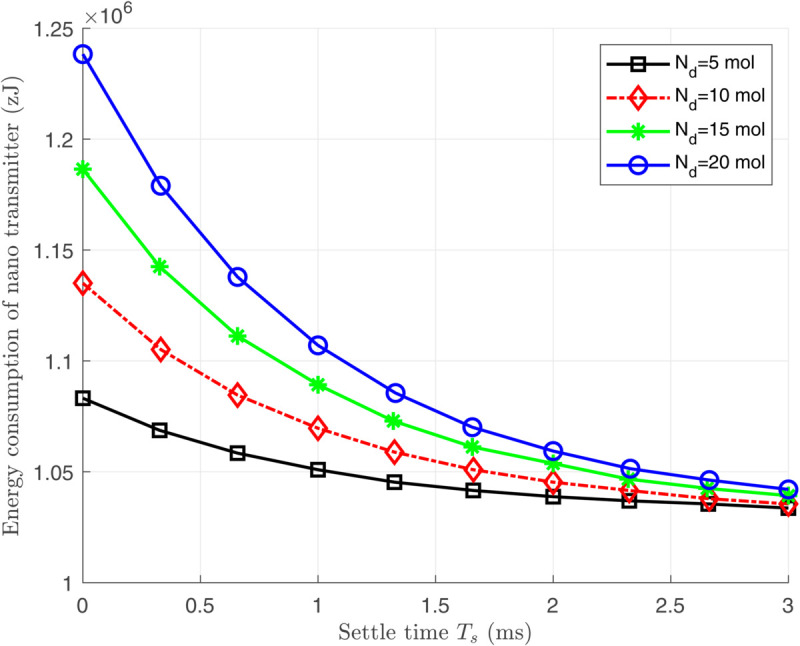
The total energy consumption *E*_*T*_ at the nano-transmitter in terms of the settle time *T*_*s*_ for different values of $N_{d}^{\left(1\right)}$.

**Fig 8:**
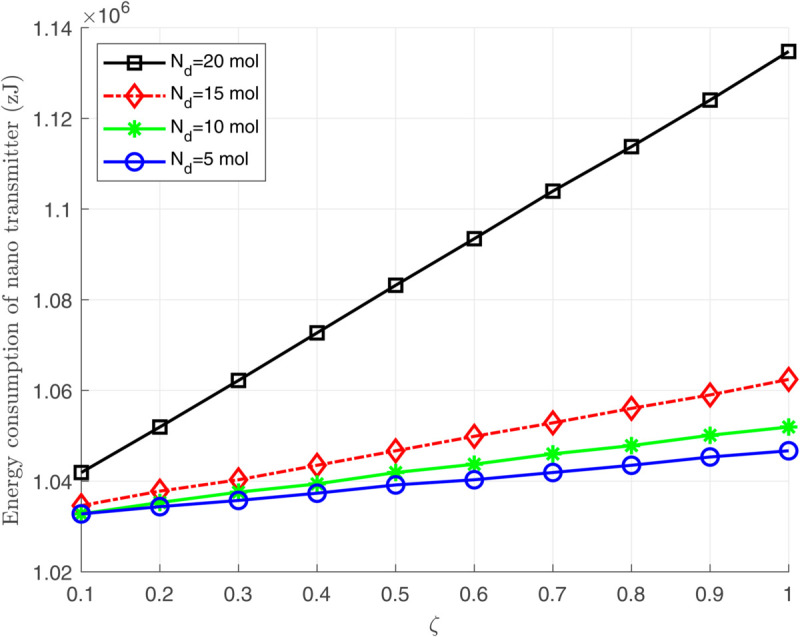
The total energy consumption *E*_*T*_ at the nano-transmitter in terms of ζ for different values of $N_{d}^{\left(1\right)}$.

Figs 6, 7, and 8 illustrate the total energy consumption at the nano-transmitter for Scenario 1 in terms of remaining molecules, *T*_*s*_, and ζ, respectively. It is observed from Fig 6 that the energy consumed at the nano-transmitter rises linearly by increasing the number of remaining molecules through the medium, as also illustrated in Eq (23). In addition, it is shown in Fig 7 that by employing the settle-time method and increasing the value of interruption period *T*_*s*_, the energy consumption at the nano-transmitter exponentially decreases. This behavior can be justified by the exponential reduction of the adverse current $\phi_{d_{total}}^{\left(n\right)}(t)$ over time (see Fig 4), leading to the exponential decrease in the energy consumption. Furthermore, as shown in Fig 8, the energy consumed at the nano-transmitter linearly increases as the parameter ζ grows. This behavior comes from the linear change in the quantity of residual molecules by changing ζ, as observed in Eq (33).

**Remark 2:** Figs 7 and 8 demonstrate that as the number of remaining molecules in the medium increases, applying the settle-time method significantly reduces the energy consumption at the nano-transmitter. This approach is particularly effective in practice when a large number of residual molecules remain in the medium after each bit transmission due to a low medium velocity.

**Fig 9:**
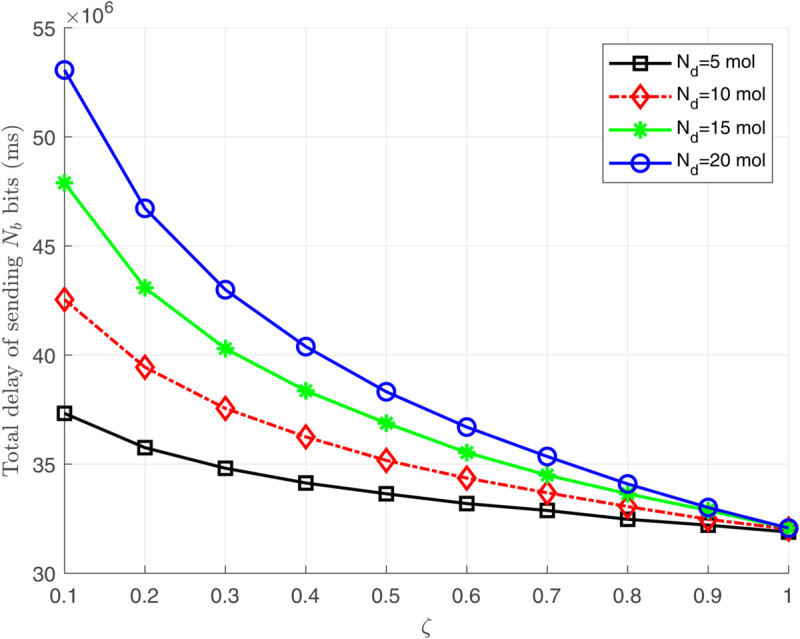
The total created delay at the nano-transmitter in terms of ζ.

**Fig 10:**
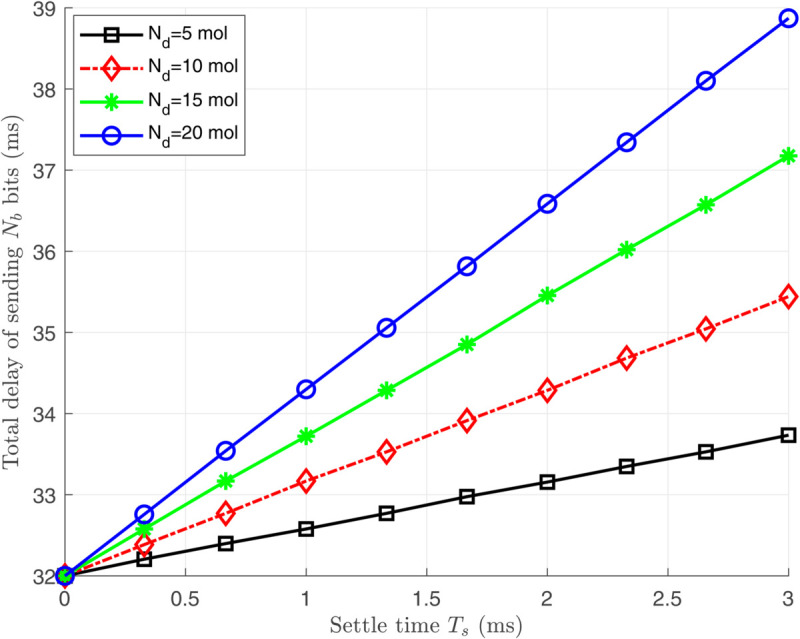
The total created delay for releasing and transmitting *N*_*p*_ bits at the nano-transmitter in terms of the settle time *T*_*s*_.

Figs 9 and 10 depict the delay at the nano-transmitter for the second scenario with varying values of ζ and *T*_*s*_. In Fig 9, it is shown that decreasing ζ for energy conservation exponentially increases the delay. Based on Eq (33), reducing ζ linearly decreases the number of remaining molecules and the adverse current $\phi_{d_{total}}^{\left(n\right)}(t)$. Consequently, considering Fig 4 and Eq (31) and Eq (36), the reduction in the adverse current $\phi_{d_{total}}^{\left(n\right)}(t)$ exponentially increases *T*_*s*_, leading to an exponential rise in delay. Furthermore, the linear relation between *T*_*s*_ and the delay is illustrated in Fig 10.

**Remark 3:** As illustrated in Fig 7, by increasing the interruption period *T*_*s*_, the medium finds enough time to counteract the effect of the remaining molecules, which reduces the energy consumed by the nano-transmitter monotonically. However, we observe from Fig 10 that the amount of the delay increases linearly by increasing the interruption period *T*_*s*_. Therefore, calculating the optimum values of ζ and *T*_*s*_ is essential for achieving proper energy consumption and delay. To clarify the clinical applicability, the modeled molecular propagation delays (32–39 ms) are well below the maximum threshold of 50 ms for glucose regulation scenarios. In addition, the delay caused by signal processing is much lower than the mechanical delay in diffusion-advection molecular communication, and can be ignored in the total delay computation. Nevertheless, additional delays from decisionmaking algorithms may contribute to the overall system delay, which is not explicitly accounted for in the current model.

**Fig 11:**
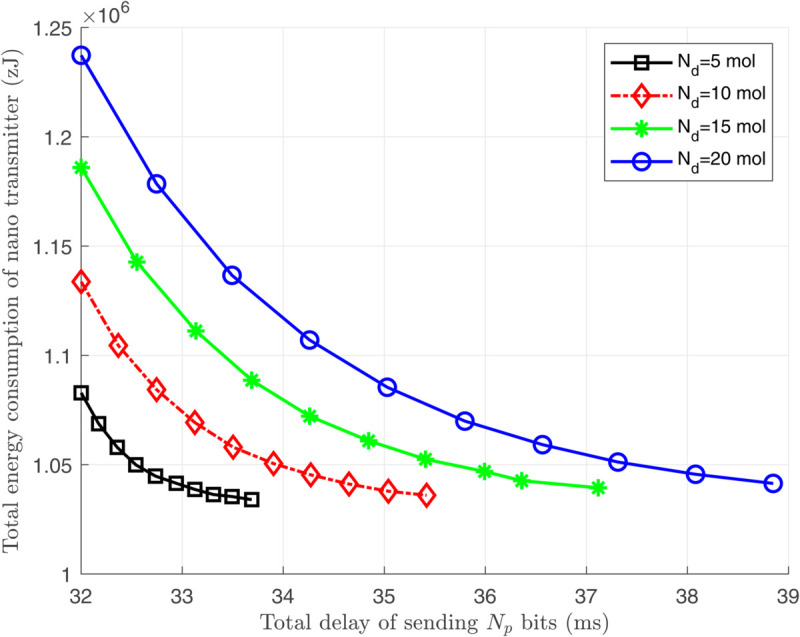
The total energy consumption *E*_*T*_ in terms of the created delay $\tau_{total}$ at the nano-transmitter.

To address the problem in Remark 3 and complete our simulation results for Scenario 3, we demonstrate the relation between the consumed energy and the created delay at the nano-transmitter in Fig 11. As shown, there is an exponential relationship between the energy consumption and delay, emphasizing that lower energy consumption can be achieved by setting small values of *T*_*s*_ at the cost of slightly increasing the delay.

## Conclusion

In this paper, we proposed a novel electrical model to capture the effect of residual molecules in the medium in an MC system. It was shown that the quantity of residual molecules after each bit transmission can be modeled as a dependent current source, generating an adverse current from the nano-receiver to the nano-transmitter. Our analysis revealed that the quantity of residual molecules resulting from the adverse current decreases monotonically over time. Additionally, we modeled the energy consumption at the nano-transmitter, demonstrating that an increase in the amount of residual molecules increases the total consumed energy at the nano-transmitter. To mitigate the negative impact of the residual molecules on energy consumption, we proposed the settle-time method and demonstrated its effectiveness through numerical results. It was shown that a trade-off exists between the length of the time interruptions in the settle-time method and the total created delay in the system, which can be controlled by adjusting the interruption period *T*_*s*_ and the parameter ζ. Finally, we concluded that utilizing small values of *T*_*s*_ can significantly reduce energy consumption, while the resulting delay at the nano-transmitter increases slightly. In this work, we performed the simulations based on Insulin molecules as the messenger molecule. Our future research involves extending the analysis and investigating how molecular properties affect the energy–delay trade-offs for a wider range of messenger molecules commonly considered for intra-body communication.
